# Coping With COVID-19: The Benefits of Anticipating Future Positive Events and Maintaining Optimism

**DOI:** 10.3389/fpsyg.2021.646047

**Published:** 2021-04-09

**Authors:** Calissa J. Leslie-Miller, Christian E. Waugh, Veronica T. Cole

**Affiliations:** ^1^Department of Psychology, William & Mary, Williamsburg, VA, United States; ^2^Department of Psychology, Wake Forest University, Winston-Salem, NC, United States

**Keywords:** coping, anticipation, optimism, positive emotion, stress, pandemic

## Abstract

In early 2020, the COVID-19 pandemic forced a large portion of the world into quarantine, leading to an extensive period of stress making it necessary to explore regulatory techniques that are effective at stimulating long-lasting positive emotion. Previous research has demonstrated that anticipating positive events produces increases in positive emotion during discrete stressors. We hypothesized that state and trait positive anticipation during the COVID-19 pandemic would be associated with increased positive emotions. We assessed how often participants thought about a future positive/negative/neutral event, activity, or goal through a daily reconstruction method that represented a “day in the life” of people in the United States during the early stages of the COVID-19 pandemic. The results of multi-level modeling and mediational analyses demonstrated that higher optimism, one form of trait positive anticipation, was related to higher state positive anticipation, which was in turn related to higher positive emotions during the current episode, which persisted to the next episode. In addition, both optimism and state positive anticipation were related to adaptive responses to the pandemic. These findings suggest that anticipation of future emotional experiences and hopefulness for the future can be a powerful predictor of positive emotions during global pandemics and perhaps other similar chronic stressors.

## Introduction

By the beginning of the year 2020, a large portion of the world was forced into quarantine by the spread of the novel COVID-19 virus, which was caused by the Severe Acute Respiratory Syndrome Coronavirus-2 (SARS-CoV2) (Andersen et al., 2020). Long periods of isolation and loneliness have been associated with increased negative emotions (Weiss, 1973), along with higher risk for health issues (Seeman, 2000; Caspi et al., 2006; Thurston and Kubzansky, 2009), hospitalization (Hastings et al., 2008), mortality (Olsen et al., 1991; Eaker et al., 1992; Sugisawa et al., 1994; Penninx et al., 1997; Shiovitz-Ezra and Ayalon, 2010), and decreased cognitive function (Cacioppo and Hawkley, 2009). With the severity of the negative effects of this extensive period of stress, it is vital to explore adaptive regulatory techniques that are effective at stimulating long-lasting positive emotions.

The COVID-19 pandemic was a chronic stressor, one that caused major disruption with no foreseeable end (Elliott and Eisdorfer, 1982). It was unique because it affected almost everyone in the world with a combination of increased minor stressors in daily life and major stressors such as sickness, financial hardship, quarantining, uncertainty, and even death (CDC, 2020). Additionally, it was a novel social stressor for most of the country, as very few people in the United States had previously experienced this type of social disconnection. This paper explores the benefits of anticipating future positive events and maintaining optimism for individuals coping with the COVID-19 pandemic in the United States during the early stages of the pandemic. State and trait positive anticipation are the focus of this paper because they are hypothesized to be effective strategies during this type of chronic stressor. In addition, although there is ample research on the role of trait positive anticipation/optimism in coping with chronic stress, there is a relative lack of research on the role of state positive anticipation for coping with chronic stress.

Experiences of positive emotion have been associated with increased well-being and improved psychological resources needed for adaptive coping (Fredrickson, 2001). Indeed, resilient responses during a stressor are characterized, in part, by the use of positive emotions (Folkman and Moskowitz, 2000). Additionally, positive emotions have been shown to be adaptive for both everyday stressors (Viney, 1986; Ong et al., 2006) and major life stressors (Fredrickson et al., 2003). Coping strategies that increase positive emotions have been found to be adaptive in treating problems such as anxiety, depression, aggression, and stress related health problems, which can be chronic in nature (Fredrickson, 2000). Daily experiences of positive emotion have been found to predict increased well-being in the months following conjugal loss (Ong et al., 2004). Taken together, it is clear that cultivating positive emotions would be an effective strategy for coping with the major and minor stressors related to the COVID-19 pandemic.

One avenue through which people experience positive emotions is through the anticipation of positive events (Van Boven and Ashworth, 2007). Anticipation involves cognitively simulating a possible future event and has been shown to accurately induce the amount of emotion that would be experienced during the event itself (Waugh et al., 2008), possibly to an even greater extent since it is novel and unanalyzed during the anticipation period (Wilson and Gilbert, 2003). Previous research on discrete stressors have demonstrated that anticipating positive events can produce increases in positive emotion both before the stressor and when recovering from the stressor (Monfort et al., 2015). The authors posited that because the positive events people are reacting to are in the future, they are able to cultivate the positive emotion associated with that anticipation, even during stressful times. This research on state positive anticipation has only focused on discrete stressors so far. However, there is reason to believe that positive anticipation could also aid the regulation of chronic stressors like the COVID-19 pandemic. The COVID-19 pandemic is a chronic stressor characterized by high levels of uncertainty throughout the population, through a variety of ways including: how it will spread, who it will affect, and when it will end (Koffman et al., 2020). Uncertainty has been found to cause increased stress responses experimentally (Miller, 1981), and naturally (Brosschot et al., 2016). When faced with uncertainty, people are naturally motivated to decrease uncertainty by gathering information (Berlyne, 1960) – a problem-focused coping strategy, but when that information is hard to come by or unreliable, they turn to other emotion-focused coping strategies (Miller, 1981). We suggest that positive anticipation is such an emotion-focused strategy that could provide boosts of positive emotion during extended times of uncertainty because it is about simulating possible future experiences and does not necessarily need to be anchored in current uncertain circumstances, such as is the case with the COVID-19 pandemic.

Additional evidence that positive anticipation may be an effective regulatory technique during a chronic stressor stems from the research on trait levels of positive anticipation as reflected by optimism. Individuals high in optimism expect good things to happen to them in the future. Previous research has found that optimism is associated with resilience (Carver et al., 2010) and predicts successful coping with significant life stressors (Scheier et al., 1986; Fredrickson et al., 2003). Optimism is adaptive when coping with uncontrollable events (Nes, 2016) and long-term stressors (Scheier and Carver, 1985). Additionally, previous research has found that individuals high in optimism are more apt to adaptively match coping strategy to the demands of the situation (Nes and Segerstrom, 2006). These individuals also experience decreased illness anxiety (Hirsch et al., 2012), decreased levels of diurnal cortisol (Jobin et al., 2014), and adaptive immune system changes as response to stress (Segerstrom and Sephton, 2010). For chronic stress, optimism has been found to be positively related to acceptance (King et al., 1998), which allows for growth in other domains (Scheier and Carver, 1992). As a form of trait positive anticipation, optimism is hypothesized to be a powerful predictor of positive emotions during the COVID-19 global pandemic.

We also sought to assess whether positive anticipation/emotions impacted some beneficial COVID-related responses. When facing high levels of uncertainty, it's important to maintain motivation to solve issues that may potentially arise. Preserving a positive and optimistic orientation to the stressor can lead to future efforts at effective problem solving (Nezu, 2004). Previous research has shown that effective problem solving reduces the negative effects of stress (Brack et al., 1992; Miner and Dowd, 1996; Cheng, 2001), therefore, we also hypothesize that positive anticipation/emotions will be positively related to people's motivation to deal with COVID-related issues. On the other hand, spending too much time thinking about a stressor with high levels of uncertainty can be problematic as the stressor itself cannot be changed. Repetitive thoughts have been found to predict increased levels of psychological distress (Smith and Alloy, 2009). It has been suggested that positive anticipation promotes successful recovery from stress in part because it replaces negative thoughts about the stressor with positive thoughts about the upcoming event (Tanner et al., 2013; Monfort et al., 2015), therefore we hypothesize that positive anticipation/emotions will be negatively related to thinking about COVID.

In this study, we assessed how often participants thought about a future positive/negative/neutral event, activity, or goal through a daily reconstruction method that represented a “day in the life” of people in the United States during the early stages of the COVID-19 pandemic (late March and April, 2020). We also measured trait positive anticipation (optimism) and its impact on emotions. The current study is a portion of a parent study that assessed coping strategy use during the early part of the COVID-19 pandemic (Waugh et al., Unpublished data). Although we assessed negative emotions and negative anticipation, positive emotions are prioritized in our hypotheses because of their importance in resilient responses to stressors (Tugade and Fredrickson, 2004).

## Materials and Methods

### Participants

Participants were recruited using Qualtrics' Panels, in which potential participants previously agreed to take part in an online panel for sharing their thoughts and opinions for research. Eligible participants were those that were over 18 years of age and resided in the US. Participants were 55.3% female and 88.4% white (*M* age = 58.27, *SD* age = 14.22). Participants were recruited to take part in a parent study (Waugh et al., unpublished data), in which they were asked to complete three surveys approximately a week apart as well as daily diaries. For this paper, we are focusing on trait optimism measured at the initial survey and the daily diary portion of this study, which took place the week after this initial survey. These data and data analyses from this paper are not presented elsewhere. The full presentation of all the surveys and measures can be found elsewhere (Waugh et al., unpublished data). Although the sample size was selected based on power analyses for effects of interest in the parent dataset, the final sample size for this study was greater than that needed (*N* = 250) to have 87% power to detect a small effect size for time-varying predictors (b = 0.2) in multilevel models when the ICC is set to 0.5 calculated in a simulation with 1,000 replications using the simr package in R (Green and MacLeod, 2015). All data and analysis scripts and surveys from both the parent study and this study are available in a data repository (https://osf.io/znjd4/?view_only=d209143537c84110b45304b77b940b0a).

Due to low retention rates typically experienced with Qualtrics, we recruited enough initial participants (*N* = 1,499) to ensure that we would have enough participants complete the full study. Participants were invited to complete up to seven daily reconstruction method (DRM: Kahneman et al., 2004) daily diary entries (see below for description). Unfortunately, participants did not complete many of these DRM daily entries with *n* = 434 completing 1, *n* = 68 completing 2, *n* = 16 completing 3, and *n* = 2 completing 4 (total *n* = 520). Since only a small percentage of participants completed more than one entry, we decided to analyze the data from each participant's first complete DRM diary entry. This analysis represents a “day in the life” of people during the early stages of the COVID-19 pandemic in the US. After excluding each participant that did not have at least one complete DRM diary entry (reported on at least one episode per time period: morning, afternoon, evening) the final DRM sample size was *n* = 329.

### Materials

#### Trait Optimism

During the initial survey, participants reported on their trait optimism using the Life Orientation Test (LOT; Scheier and Carver, 1985) on a scale from 1 (strongly disagree) to 5 (strongly agree); α = 0.92.

#### Daily Diary

For the week after the initial survey, participants completed the daily diary entry at the end of each day – sometime after dinner but before bedtime. They were told that we were interested in what they did and how they felt that day. They were asked to reconstruct their day as if they were writing in a diary (Kahneman et al., 2004). Although retrospective, this method has been shown to accurately capture the emotional dynamics of daily life including how emotions at one time point impact emotions at another time point (Waugh et al., 2017). They described what happened for each episode that occurred in the morning, afternoon, and evening (up to 10 for each time frame for a possible total of up to 30) and what time it began and ended. An episode was included in the analyses if there was no more than one missing value for participants' subsequent ratings of that episode (*M* episodes = 11.2, *SD* = 5.74).

#### State Anticipation

For each episode, participants reported on how often during that episode they thought about a future positive/negative/neutral “event, activity, or goal” from 1 (not at all) to 4 (very often). These were single items because participants had to report these anticipatory thoughts on every episode of the day.

#### State Emotions

Participants rated their emotions during that episode on a 0 (not at all) to 6 (very) scale. They reported on their stress, control (of their feelings), pleasantness (positive emotions), and unpleasantness (negative emotions).

#### COVID-Related Issues

Participants also rated how often during that episode they thought about the coronavirus from 0 (not at all) to 6 (very) and how motivated they would be to engage in some activity related to dealing with issues caused by the coronavirus pandemic from 1 (not motivated at all) to 4 (very motivated).

### Analyses

We first conducted bivariate correlations among trait optimism, mean levels of positive, negative, neutral anticipation as well as mean levels of the emotional outcomes (positive/negative emotions, stress, control) and the COVID-related outcomes (thinking about COVID, motivated to deal with COVID). To adjust for multiple comparisons, we applied the Benjamini-Hochberg correction (Benjamini and Hochberg, 1995) with the number of correlation tests set to 45. We note which findings did not survive multiple comparison correction.

We next conducted exploratory mediation analyses to more fully flesh out possible relationships among optimism, positive/negative anticipation, positive/negative emotions, and the COVID-related variables. We used PROCESS (Hayes, 2013), an SPSS macro, to calculate indirect and direct effects.

Lastly, we conducted separate multilevel models (MLM; with the R package lme4; Bates et al., 2015) with episode anticipation (positive, neutral, negative) as a person-centered predictor of episode-related positive emotions, negative emotions, stress, control of feelings, thinking about COVID, and motivation to deal with COVID. We first conducted a set of models testing the relationships between **concurrent** anticipation and outcomes (i.e., *anticipation*_*e*1_ -> *outcome*_*e*1_) to determine whether anticipating a future event affects current outcomes. We next conducted another set of models testing the **lagged** relationships between anticipation at one episode and outcomes at the next episode (i.e., *anticipation*_*e*1_ -> *outcome*_*e*+1_) to determine whether anticipating a future event affects subsequent outcomes. Lastly, we conducted a set of models testing **lagged with autocorrelation** relationships between anticipation at one episode and outcomes at the next episode controlling for autocorrelations by specifying an AR(2) structure for the error variance-covariance matrix (to account for effects of the prior two episodes) in the MLM models using the R package nlme (Pinheiro et al., 2021). These models test whether anticipation predicts outcomes in subsequent episodes above and beyond the effects of the outcomes from prior episodes. For all of the models, along with person-centered predictors, we also included each person's mean levels of the predictor at Level 1 nested within participant at Level 2. The mean levels of predictors allowed us to differentiate within-participant effects (e.g., concurrent, lagged) of anticipation on outcomes from between-participant effects of anticipation on outcomes. We report only the between-participant effects from the concurrent model because these included all of the outcome reports (vs. lagged when they included n−1 reports) and are therefore most comparable to the between-participant correlations. To adjust for multiple comparisons, we again applied the Benjamini-Hochberg correction with the number of tests set to 18 (3 predictors × 6 outcome variables for each effect of interest). We note which findings did not survive multiple comparison correction. Notably, controlling for age or gender at level 2 did not change any of the findings.

## Results

### Relationships Among Optimism, Anticipation, and Emotional Outcomes

#### Correlations

Supporting our hypothesis, trait optimism and state positive anticipation were positively related to positive emotions (Table 1). Also consistent with our hypotheses, positive emotions were negatively related to thinking about COVID and positively related to motivation to deal with COVID (which were not correlated with each other).

**Table 1:** Descriptives and correlations among the variables of interest.

			**Correlations**								
	**Mean (SD)**	**ICC**	**1**	**2**	**3**	**4**	**5**	**6**	**7**	**8**	**9**
1. Optimism	3.6 (0.87)	–	–								
2. Positive anticipation	2.32 (0.88)	0.64	0.159^**^	–							
3. Neutral anticipation	1.9 (0.71)	0.64	−0.007	0.596^**^	–						
4. Negative anticipation	1.54 (0.66)	0.60	−0.289^**^	0.112^*^	0.497^**^	–					
5. Positive Emotions	4.11 (1.35)	0.55	0.336^**^	0.316^**^	0.110^*^	−0.229^**^	–				
6. Negative Emotions	1.38 (1.44)	0.60	−0.335^**^	−0.014	0.291^**^	0.737^**^	−0.367^**^	–			
7. Stress	1.35 (1.53)	0.62	−0.327^**^	0.067	0.314^**^	0.679^**^	−0.324^**^	0.899^**^	–		
8. Control	4.74 (1.35)	0.64	0.313^**^	0.211^**^	0.032	−0.308^**^	0.795^**^	−0.409^**^	−0.434^**^	–	
9. Think about COVID	2.09 (1.53)	0.52	−0.251^**^	0.099	0.332^**^	0.591^**^	−0.249^**^	0.747^**^	0.728^**^	−0.303^**^	–
10. Motivated to deal with COVID	2.5 (0.87)	0.62	0.253^**^	0.437^**^	0.293^**^	0.009	0.428^**^	−0.126^*^	−0.085	0.325^**^	0.049

** *p corrected < 0.05*,

* *p uncorrected < 0.05*.

More generally, trait optimism was related to an overall positive profile – lower state negative anticipation, lower negative emotions and stress, higher control and motivation to deal with COVID and less thinking about COVID (Table 1). Positive anticipation was also positively related to control and motivation to deal with COVID. Negative anticipation featured an overall negative profile that was almost exactly opposite to that of trait optimism with the exception that there was not a significant relationship between negative anticipation and motivation to deal with COVID.

Strangely, neutral anticipation was highly correlated with both positive and negative anticipation (which were only mildly related to each other) and therefore exhibited a mixed pattern of relationships with outcomes such as higher positive (uncorrected) and negative emotions/stress and more thinking about COVID but also being motivated to deal with COVID.

#### Mediations

##### Positive Anticipation/Emotion

**Figure 1:**
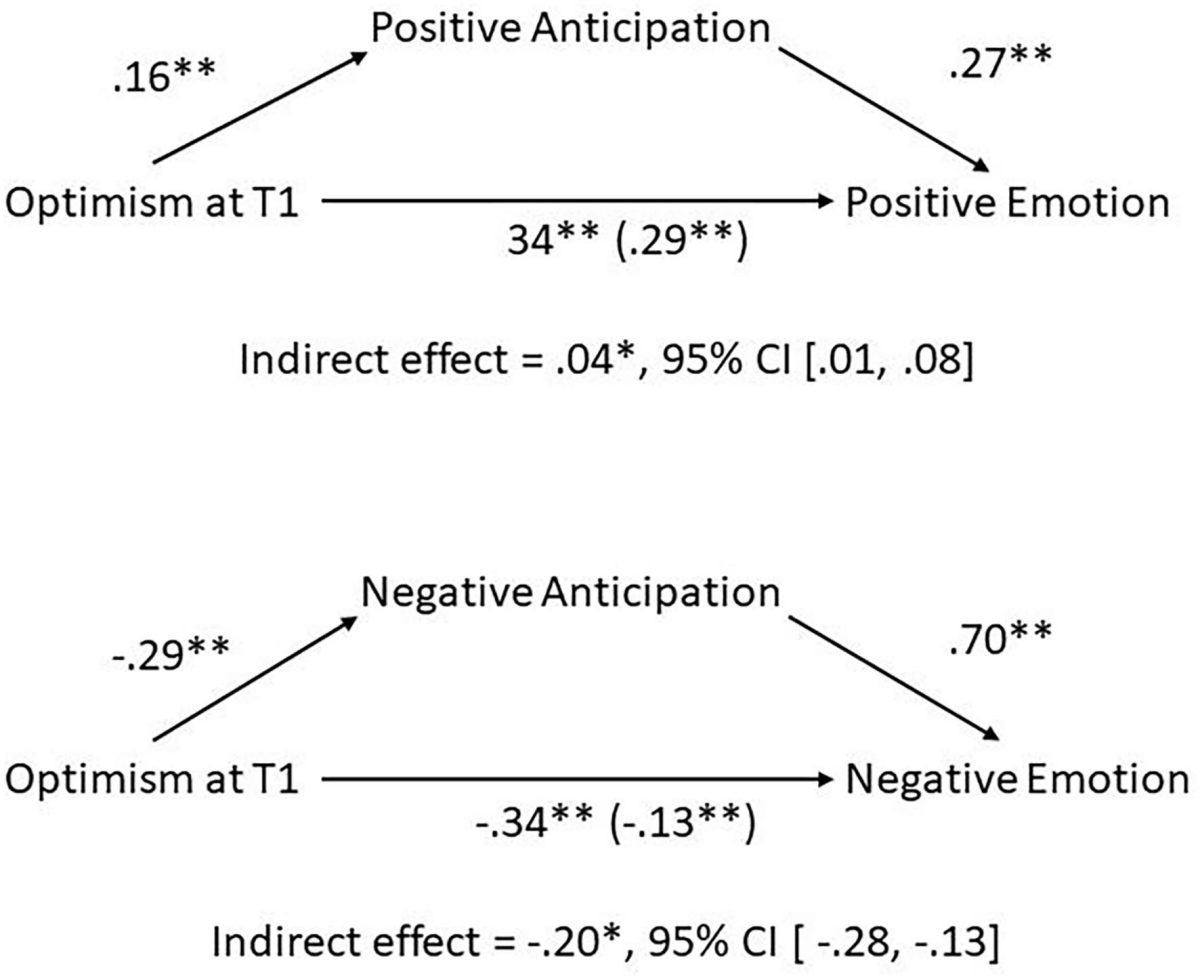
Mediational relationships among trait optimism measured at T1 survey and daily reports of positive/negative anticipation and positive/negative emotions. Effects are standardized. *p < 0.05, **p < 0.01.

Daily positive anticipation partially mediated the relationship between trait optimism and positive emotion (Figure 1). In addition, using both positive anticipation and positive emotion as serial mediators, they partially mediated the relationship between optimism and thinking about COVID and between optimism and motivation to deal with COVID (Table 2). Therefore, optimistic people more often anticipated positive events, which in turn led to more daily positive emotions, which in turn led to more beneficial responses to COVID.

**Table 2:** Mediational models with optimism predicting emotions through anticipation.

**Outcome → **	**Motivated to deal with COVID issues**				**Think about COVID**			
**Predictor/Mediators** **↓**	**Direct effect (SE)**	**95% CI**	**Indirect effect (SE)**	**95% CI**	**Direct effect (SE)**	**95% CI**	**Indirect effect (SE)**	**95% CI**
Optimism	0.10 (0.05)	0.01, 0.20			−0.35 (0.10)	−0.54, −0.16		
→ Pos Ant			0.05 (0.02)	0.01, 0.10			0.06 (0.03)	0.01,0.11
→ Pos Emo			0.08 (0.02)	0.05, 0.13			−0.13 (0.04)	−0.21, −0.05
→ Pos Ant			0.01 (0.01)	0.00, 0.03			−0.02 (0.01)	−0.04, −0.00
→ Pos Emo								
Optimism	0.25 (0.06)	0.14, 0.36			0.01 (0.07)	−0.13, 0.14		
→ Neg Ant			−0.07 (0.03)	−0.13, −0.02			−0.05 (0.05)	−0.15, 0.04
→ Neg Emo			0.03 (0.01)	0.01, 0.06			−0.16 (0.05)	−0.26, −0.07
→ Neg Ant			0.05 (0.02)	0.01, 0.08			−0.25 (0.06)	−0.38, −0.14
→ Neg Emo								

*Effects are unstandardized. SE, standard error; CI, confidence interval; Ant, anticipation; Pos, positive; Neg, negative; Emo, emotion*.

##### Negative Anticipation/Emotion

Daily negative anticipation partially mediated the relationship between trait optimism and negative emotion (Figure 1). Also, as serial mediators, negative anticipation and negative emotions partially mediated the relationship between optimism and motivation to deal with COVID, and fully mediated the relationship between optimism and thinking about COVID (Table 2). These findings mirror the ones found above for positive anticipation except that the link between increased trait optimism and decreased thinking about COVID was fully mediated by decreased negative anticipation and negative emotions.

### Multilevel Modeling of the Relationships Between Anticipation and Emotional Outcomes

#### Positive Anticipation

Consistent with hypotheses, positive anticipation was a significant predictor of concurrent levels of higher positive emotion, control, and motivation to deal with COVID as well as lower negative emotion and stress (Table 3). Positive anticipation during one episode also predicted higher positive emotions, control and motivation to deal with COVID at the next episode, however it only predicted control during the next episode when accounting for autocorrelations. Positive anticipation was once again unrelated to thinking about COVID.

**Table 3:** Multilevel models of relationship between future thinking and emotional responses during daily diary events.

**X↓/Y →**	**PE**	**NE**	**Str**	**CTL**	**Think COVID**	**Motiv COVID**
**Future positive**						
Between-participants	0.08	0.08	0.12^*^	0.03	0.06	0.23^**^
Concurrent	0.21^**^	−0.1^**^	−0.07^**^	0.17^**^	0.02	0.14^**^
Lagged	0.07^**^	0	0.01	0.09^**^	0.03	0.06^**^
Lagged controlling for autocorrelation	0.05^*^	0.01	0.01	0.06^**^	0.3	0.03
**Future neutral**						
Between-participants	0.05	0.2^**^	0.23^**^	−0.07	0.14^**^	0.18^**^
Concurrent	0.05^**^	0.07^**^	0.06^**^	0.11^**^	0.14^**^	0.06^**^
Lagged	0.05^*^	0.03	0.05^*^	0.05^*^	0.02	0.05^**^
Lagged controlling for autocorrelation	0.05^*^	0.02	0.04^*^	0.03	0.00	0.03
**Future negative**						
Between-participants	−0.03	0.33^**^	0.31^**^	−0.2^**^	0.18^**^	0.01
Concurrent	−0.19^**^	0.34^**^	0.3^**^	−0.07^**^	0.33^**^	0
Lagged	0.01	0.08^**^	0.08^**^	0	0.06^**^	−0.01
Lagged controlling for autocorrelation	0.02	0.05^*^	0.05^*^	−0.01	0.02	−0.01

*Standardized betas are shown. PE, positive emotion; NE, negative emotion; Str, stress; CTL, control; Motiv, motivation. Between-participants' effects for mean predictors are shown for the model including the concurrent (within-participants) predictor of X on Y, but mean predictors were also controlled for in the other models (lagged, lagged controlling for autocorrelation)*.

** *p corrected < 0.05*,

* *p uncorrected < 0.05*.

#### Negative Anticipation

Negative anticipation exhibited a pattern of relationships that was largely opposite to that of positive anticipation. It was related to lower concurrent positive emotions and control and higher concurrent negative emotions, stress and thinking about COVID. Negative anticipation at the current episode also predicted increased negative emotions, stress, and thinking about COVID at the next episode, however none of these relationships remained when controlling for autocorrelations.

#### Neutral Anticipation

Similar to the between-subject correlations, there was a mixed pattern of relationships between neutral anticipation and concurrent emotional outcomes in that it was related to higher levels of all the outcomes. The only lagged relationship that survived correction was that neutral anticipation during one episode predicted increased motivation to deal with COVID at the next episode, however, this relationship did not remain significant (corrected) when controlling for autocorrelations.

## Discussion

This study aimed to demonstrate that state and trait positive anticipation are effective at increasing positive emotions during the COVID-19 pandemic. Positive anticipation and optimism were both found to predict increases in positive emotions concurrently. In addition, the more participants engaged in positive anticipatory thinking during one episode, the more they experienced positive emotions at the next episode of their daily lives. Because positive anticipation did not also predict changes in positive emotions from one episode to the next controlling for autocorrelations, this pattern of findings suggests that positive anticipation helps people feel good in the moment and that these current positive emotions may persist to subsequent activities rather than positive anticipation generating subsequent positive emotions unrelated to its effect on current positive emotions. Importantly, the relationships between positive anticipation and positive emotions were strong even though people were in the midst of the COVID-19 pandemic, which extends previous research that anticipating positive events can produce increases in positive emotion to discrete stressors in response to stressors (Monfort et al., 2015) to also include chronic, all-encompassing stressors. Similarly, optimism was found to predict positive emotions during this uncontrollable and persistent stressor which supports previous research on optimism (Scheier and Carver, 1985; Nes, 2016), and this relationship was partially mediated by positive anticipation throughout the day. This finding supports the idea that optimism works as a trait-level predictor of positive anticipation, but also leaves open the possibility that optimism predicts positive emotions through other mechanisms as well or that we did not fully capture optimism-related positive anticipation in our daily diaries.

The above findings suggest that state and trait positive anticipation can predict positive emotions during a stressor, but we also sought to demonstrate that they were important for dealing with COVID specifically. Consistent with our hypotheses, positive emotions were negatively related to thinking about COVID. Repetitive thoughts about uncontrollable stressors have been found to predict increased levels of psychological distress (Smith and Alloy, 2009), so this finding suggests that experiencing positive emotions may replace those negative repetitive thoughts (Quoidbach et al., 2010). Furthermore, our findings add to previous research on the negative relationship between optimism and rumination (repetitive and intrusive thinking about negative emotions and events; Tucker et al., 2013), by showing that this relationship may be due to optimists anticipating positive, but not negative events and experiencing positive, but not negative emotions.

Previous research has shown that effective problem solving reduces the negative effects of stress (Brack et al., 1992; Miner and Dowd, 1996; Cheng, 2001), which highlights the importance of being motivated to deal with the problems associated with a chronic stressor such as COVID-19. Mirroring the above findings for thinking about COVID, we found that positive emotions were positively related with the motivation to deal with COVID, which supports the roles of positive emotions as motivators of adaptive behavior (Fritz and Sonnentag, 2007; Løvoll et al., 2017). Again, optimism and positive anticipation were also both related to motivation to deal with COVID through their relationship with positive emotions. Part of the power of positive anticipation is that it gives people something to look forward to and increases motivation to obtain that anticipated thing (Løvoll et al., 2017) – we showed that this motivation may also carry-over to dealing with the more unpleasant aspects of a chronic stressor.

Due to possible cultural differences in how people value emotions, this study can only generalize to individuals in the United States during the COVID-19 pandemic. Studies comparing cross-cultural differences in how people responded to this worldwide pandemic are needed, especially given cultural differences in the importance of high arousal positive emotions (Tsai, 2007), which usually accompany heightened positive anticipation. Additionally, all data were collected during the pandemic without a pre-pandemic baseline. Due to this limitation, we cannot determine whether these relationships change as a result of being in the COVID-19 pandemic or not. Additionally, for whatever reason, our recruitment methods resulted in an older sample than we intended. Although this is good for showing coping in those most vulnerable to the ill effects of the coronavirus (National Center for Health Statistics, 2020), it does suggest that we cannot fully generalize these findings to a younger sample.

## Summary

The COVID-19 global pandemic was a novel chronic stressor that severely impacted the United States, along with the majority of the world. Positive anticipation and optimism were effective strategies for coping with COVID-19 because they increased positive emotion. These findings suggest that anticipation of future emotional experiences and optimism for the future can be a powerful predictor of positive emotions during global pandemics and perhaps other similar chronic stressors that severely disrupt daily life, feature high levels of uncertainty, lead to increased isolation and loneliness, and do not have a foreseeable end. This study adds to the literature for adaptive coping with the COVID-19 pandemic, and uniquely explores the adaptive role of state and trait positive anticipation for a chronic stressor.

## Data Availability Statement

The datasets presented in this study can be found in online repositories. The names of the repository/repositories and accession number(s) can be found below: https://osf.io/znjd4/?view_only=d209143537c84110b45304b77b940b0a.

## Ethics Statement

The studies involving human participants were reviewed and approved by Wake Forest University Institutional Review Board. Participants provided their informed consent to participate in the study.

## Author Contributions

CL-M, CW, and VC were involved in the design of the study. CL-M and CW were involved in conducting the study. CW and VC were involved in data analysis. All authors were involved in writing the manuscript and approved the submitted version.

## Conflict of Interest

The authors declare that the research was conducted in the absence of any commercial or financial relationships that could be construed as a potential conflict of interest.
